# Supplementary material for: Formononetin inhibits lipopolysaccharide-induced release of high mobility group box 1 by upregulating SIRT1 in a PPARδ-dependent manner
Source: PeerJ. 2018 Jan 3;6:e4208. doi: 10.7717/peerj.4208 (PMC5756453; doi:10.7717/peerj.4208)
Supplement: Supplemental Information 1 — Raw and statistical data of MTT assay for Fig. 1A. Raw and statistical data of Trypan blue exclusion assay for Fig. 1B. Raw and statistical data for the ratios of HMGB1 to each control of Fig. 1D. Raw and statistical data for the ratios of HMGB1 to each control of Fig. 1E. Raw and statistical data for the ratios of HMGB1 to each control of Fig. 1F. Raw and statistical data of real-time pcr for Fig. 1G. [file peerj-06-4208-s001.pdf]

RAW data

Figure 1A

dose (μM)	0	1	5	10	20	30	40	50
n=1	2.083	2.19	2.085	2.036	2.183	2.169	1.671	1.36
n=2	2.122	1.922	2.156	2.133	2.075	1.873	1.591	1.475
n=3	2.078	2.091	2.191	2.081	2.048	1.916	1.705	1.31
n=4	2.034	1.903	1.981	2.388	2.184	2.083	1.567	1.486
average	2.0793	2.0265	2.1033	2.1595	2.1225	2.0103	1.6335	1.4077
S.E	0.018	0.069	0.0463	0.0787	0.0356	0.0697	0.0326	0.0433

dose (μM)	0	1	5	10	20	30	40	50
n=1	100	105.1368	100.096	97.74364	104.8008	104.1287	80.22084	65.29045
n=2	100	90.57493	101.6023	100.5184	97.78511	88.26579	74.97644	69.5099
n=3	100	100.6256	105.4379	100.1444	98.5563	92.20404	82.05005	63.04139
n=4	100	93.55949	97.3943	117.4041	107.3746	102.409	77.04031	73.05801
average	100	97.4742	101.1326	103.9526	102.1292	96.7519	78.5719	67.7249
S.E	0	3.3112	1.6784	4.5258	2.3503	3.8635	1.5835	2.2265

RAW data

Figure 1B

formonone	0day	1day	2day	3day	4day	5day
N=1	30	28	32	28	25	26
N=2	28	26	22	20	21	31
N=3	29	27	28	28	26	31
N=4		24	30	31	31	19

RAW data

Figure 1D

	HMGB1	Ponceau S	HMGB1/Pc	fold
1	12677.82	30234.8	0.419312	1
2	41014.84	31755.34	1.291589	3.080256
3	18089.63	31993.7	0.565412	1.348428
4	23726.55	39990.38	0.593306	1.414951

	HMGB1	Ponceau S	HMGB1/Pc	fold
1	6278.409	46715.68	0.134396	1
2	30828.62	48447.78	0.636327	4.734709
3	14343.46	45536.44	0.314989	2.343732
4	8813.794	49656.51	0.177495	1.320687

	HMGB1	Ponceau S	HMGB1/Pc	fold
1	13365.46	46851.75	0.285271	1
2	40760.84	47057.15	0.866199	3.036404
3	13909.41	44047.53	0.315782	1.106953
4	2034.196	44934.8	0.04527	0.158691

	HMGB1	actin	HMGB1/ac	fold
1	38472.65	38137.53	1.008787	1
2	39365.2	42261.29	0.931472	0.923358
3	43609.41	39790.24	1.095983	1.086436
4	45760.8	33738.53	1.356337	1.344522

	HMGB1	actin	HMGB1/ac	fold
1	42066.58	37185.41	1.131266	1
2	46486.8	40588.53	1.145319	1.012422
3	44792.34	39388.7	1.137187	1.005235
4	44417.77	41937.97	1.05913	0.936235

	HMGB1	actin	HMGB1/ac	fold
1	42535.07	35078.65	1.212563	1
2	40919.75	39692.95	1.030907	0.850189
3	41512.34	41850.7	0.991915	0.818032
4	42225.82	42726.48	0.988282	0.815036

RAW data

Figure 1E

	hmgb1	PonS	HMGB1/pc	fold
1	5469.388	47185.7	0.115912	1
2	39408.02	47990.51	0.821163	7.084364
3	10747.66	47366.15	0.226906	1.957571
4	7444.338	47556.56	0.156537	1.350477

	hmgb1	PonS	HMGB1/pc	fold
1	1594.255	21307.44	0.074822	1
2	28492.5	32422.65	0.878784	11.74507
3	9822.217	33763.22	0.290915	3.888116
4	2911.631	34339.41	0.08479	1.133227

	hmgb1	PonS	HMGB1/pc	fold
1	6879.267	49834.24	0.138043	1
2	37128.72	43149.85	0.86046	6.233276
3	7212.823	40613.63	0.177596	1.286528
4	6614.388	45025	0.146905	1.064196

	hmgb1	actin	HMGB1/ac	fold
1	35637.07	37289.07	0.955697	1
2	34508.99	39049.36	0.883727	0.924694
3	38863	35183.63	1.104576	1.15578
4	34194.19	37401.07	0.914257	0.956639

	hmgb1	actin	HMGB1/ac	fold
1	34741.31	46003.45	0.755189	1
2	31262.39	32462.66	0.963026	1.275212
3	36871.07	41477.51	0.888941	1.177111
4	33814.89	40348.65	0.838068	1.109745

	hmgb1	actin	HMGB1/ac	fold
1	31307.12	44109.56	0.709758	1
2	36330.87	42945.95	0.845967	1.191909
3	37568.7	43542.25	0.86281	1.21564
4	39679.65	42395.7	0.935936	1.318669

RAW data

Figure 1F

	HMGB1	laminB&tu	HMGB1/lo	fold
1	17367.35	13231.15	1.31261	1
2	2229.891	16404.15	0.135935	0.10356
3	18053.15	18909.69	0.954704	0.727332
4	12204.96	17296.4	0.705636	0.537582
5	20399.28	16626.98	1.226878	0.934686
6	2632.305	15384.74	0.171098	0.13035
7	18483.98	13051.86	1.416195	1.078915
8	2958.326	21775.81	0.135854	0.103499

	HMGB1	laminB&tu	HMGB1/lo	fold
1	12199.5	16270.91	0.749773	1
2	6118.598	17908.23	0.341664	0.45569
3	18920.69	20235.98	0.935002	1.247046
4	9824.062	16628.86	0.590784	0.78795
5	20113.88	17130.3	1.17417	1.566033
6	4964.962	15582.74	0.318619	0.424954
7	8904.569	13096.52	0.679919	0.906832
8	4282.255	20551.98	0.208362	0.2779

	HMGB1	laminB&tu	HMGB1/lo	fold
1	17162.81	14448.98	1.187821	1
2	1925.77	17943.52	0.107324	0.090354
3	15243.15	16481.45	0.924867	0.778625
4	10996.55	16381.38	0.671284	0.565138
5	21574.76	18399.15	1.172595	0.987181
6	2619.113	20401.52	0.128378	0.108079
7	21994.98	15849.1	1.387775	1.168336
8	2610.79	21620.24	0.120757	0.101662

RAW data

Figure 1G

mHMGB1

Replicate N	GOI CT	Norm. CT	Relative Cc	Calibrator
1	21.82	16.22	1	Yes
2	21.55	15.98	1.03	
3	22.02	16.31	0.96	
4	21.8	15.61	1.03	

Replicate N	GOI CT	Norm. CT	Relative Cc	Calibrator
1	21.36	16.22	1	Yes
2	21.66	15.98	0.91	
3	21.24	16.31	1.04	
4	20.8	15.61	0.96	

Replicate N	GOI CT	Norm. CT	Relative Cc	Calibrator
1	21.33	16.22	1	Yes
2	21.06	15.98	0.99	
3	21.08	16.31	0.96	
4	20.8	15.61	0.94	

Replicate N	GOI CT	Norm. CT	Relative Cc	Calibrator
1	21.84	16.22	1	Yes
2	21.78	15.98	0.96	
3	21.04	16.31	1.04	
4	21.8	15.61	1.01	

n=1	1	1.03	0.96	1.03
n=2	1	0.91	1.04	0.96
n=3	1	0.99	0.96	0.94
n=4	1	0.96	1.04	1.01
average	1	0.9725	1	0.985
S.E	0	0.0253	0.0231	0.021
