# Supplementary material for: Formononetin inhibits lipopolysaccharide-induced release of high mobility group box 1 by upregulating SIRT1 in a PPARδ-dependent manner
Source: PeerJ. 2018 Jan 3;6:e4208. doi: 10.7717/peerj.4208 (PMC5756453; doi:10.7717/peerj.4208)
Supplement: Supplemental Information 2 — Raw and statistical data for the ratios of SIRT1 to β-actin of Fig. 2A. Raw and statistical data for the ratios of SIRT1 to β-actin of Fig. 2B. Raw and statistical data of real-time pcr for Fig. 2C. Raw and statistical data of real-time pcr for Fig. 2E. Raw and statistical data of luciferase assay for Fig. 2H. [file peerj-06-4208-s002.pdf]

RAW data

Figure 2A

formononetin dose

	SIRT1	actin	SIRT1/actir	fold
1	12415.18	23929.3	0.518827	1
2	14282.71	24157.83	0.591225	1.139541
3	16164	20642.18	0.783057	1.509283
4	18562.42	22417.59	0.828029	1.595963
5	22874.95	23047	0.992535	1.913035
6	25619.15	26142.9	0.979966	1.888808

formononetin dose

	SIRT1	actin	SIRT1/actir	fold
1	9424.368	32132.61	0.293296	1
2	17190.25	29431.18	0.584083	1.991445
3	24335.3	29219.18	0.832854	2.839635
4	24481.25	28565.47	0.857022	2.922039
5	26794.78	29297.64	0.914571	3.118253
6	25423.02	30709.54	0.827854	2.822589

formononetin dose

	SIRT1	actin	SIRT1/actir	fold
1	11341.47	29196.95	0.388447	1
2	11135.37	27134	0.410384	1.056474
3	15032.83	27304.64	0.55056	1.417335
4	16891.93	27306.57	0.618603	1.592504
5	27878.08	27216.35	1.024314	2.636946
6	24166.42	25452.42	0.949474	2.444283

RAW data

Figure 2B

formononetin TIME

	SIRT1	actin	SIRT1/actir	fold
1	9812.811	18992.71	0.516662	1
2	9538.953	22412	0.425618	0.823784
3	10410.64	21907.42	0.475211	0.919771
4	12356.83	24244.83	0.509669	0.986465
5	20615.71	25289	0.815205	1.57783
6	27581.59	28677.66	0.96178	1.861526

formononetin TIME

	SIRT1	actin	SIRT1/actir	fold
1	12097.18	24298.95	0.497848	1
2	16068.88	25282.83	0.635565	1.276626
3	21460.3	26271.42	0.816869	1.640801
4	25133.47	21920.23	1.146588	2.303091
5	27389.93	29883.42	0.91656	1.841045
6	23521.42	23802.83	0.988177	1.984899

formononetin TIME

	SIRT1	actin	SIRT1/actir	fold
1	8024.569	26529.22	0.30248	1
2	12981.3	24280.81	0.534632	1.767493
3	15290.83	25678.81	0.595465	1.968607
4	13729.3	28641.05	0.479357	1.584754
5	21474.66	29630.98	0.724737	2.395979
6	25405	31639.13	0.802962	2.65459

RAW data

Figure 2C

n=1	1	0.75	1.7	2.14	3.8	4.52
n=2	1	0.94	1.86	3.23	3.7	3.96
n=3	1	1.03	1.2	2.38	4.02	4.12
average	1	0.9067	1.5867	2.5833	3.84	4.2
S.E	0	0.0825	0.1988	0.3307	0.0945	0.1665

RAW data

Figure 2E

n=1	1	3.97	2.01	1.48	0.92	0.48
n=2	1	2.28	1.31	1.35	1.01	0.89
n=3	1	3.5	1.03	1.09	0.24	1.02
average	1	3.25	1.45	1.3067	0.7233	0.7967
S.E	0	0.5036	0.2914	0.1146	0.2431	0.1627

RAW data

Figure 2H

	SIRT1 lucif	B-gal	lucifer/b-g	Fold
1	8241	0.102	80794.12	1
2	27532	0.118	233322	2.887859
3	16022	0.111	144342.3	1.786545
4	9880	0.112	88214.29	1.09184

	SIRT1 lucif	B-gal	lucifer/b-g	Fold
11	16056	0.121	132694.2	1
12	34215	0.114	300131.6	2.261829
13	21255	0.117	181666.7	1.369062
14	10616	0.108	98296.3	0.740773

	SIRT1 lucif	B-gal	lucifer/b-g	Fold
21	16524	0.122	135442.6	1
22	29442	0.109	270110.1	1.994277
23	19394	0.113	171628.3	1.267166
24	9299	0.104	89413.46	0.660157
