# Supplementary material for: Formononetin inhibits lipopolysaccharide-induced release of high mobility group box 1 by upregulating SIRT1 in a PPARδ-dependent manner
Source: PeerJ. 2018 Jan 3;6:e4208. doi: 10.7717/peerj.4208 (PMC5756453; doi:10.7717/peerj.4208)
Supplement: Supplemental Information 3 — Raw and statistical data for the ratios of SIRT1 and HMGB1 to each control of Fig. 3A. Raw and statistical data for the ratios of HMGB1 to Ponceau S of Fig. 3C. Raw and statistical data for the ratios of HMGB1 to Ponceau S of Fig. 3D. Raw and statistical data for the ratios of HMGB1 to Ponceau S of Fig. 3E. [file peerj-06-4208-s003.pdf]

RAW data

**Figure 3A**

| | SIRT1 | actin | SIRT1/actir | fold | | SIRT1 | actin | SIRT1/actir | fold | | SIRT1 | actin | SIRT1/actir | fold |
|---|---|---|---|---|---|---|---|---|---|---|---|---|---|---|
| 1 | 18267.24 | 27602.95 | 0.661786 | 1 | 1 | 23013.24 | 42677.58 | 0.539235 | 1 | 1 | 32888.79 | 36529.19 | 0.900343 | 1 |
| 2 | 4303.468 | 38260.14 | 0.112479 | 0.169963 | 2 | 7228.61 | 38786.92 | 0.186367 | 0.345614 | 2 | 10908.82 | 37334.27 | 0.292193 | 0.324536 |
| 3 | 14961.95 | 25391.41 | 0.589252 | 0.890397 | 3 | 18285.41 | 38581.22 | 0.473946 | 0.878923 | 3 | 27113.02 | 46437.8 | 0.583857 | 0.648482 |
| 4 | 44390.44 | 27230.48 | 1.630175 | 2.463296 | 4 | 37144.55 | 38434.31 | 0.966443 | 1.792248 | 4 | 46399.29 | 32001.87 | 1.449893 | 1.610378 |

| | LPS+Formononetin: HMGB1 | | | | | LPS+Formononetin: HMGB1 | | | | | LPS+Formononetin: HMGB1 | | | |
|---|---|---|---|---|---|---|---|---|---|---|---|---|---|---|
| | HMGB1 | PonS | HMGB1/Pc | fold | | HMGB1 | PonS | HMGB1/Pc | fold | | HMGB1 | PonS | HMGB1/Pc | fold |
| 1 | 5906.024 | 39339.7 | 0.150129 | 1 | 1 | 12686.7 | 29949 | 0.42361 | 1 | 1 | 8215.752 | 24192.29 | 0.339602 | 1 |
| 2 | 38088.29 | 43027.7 | 0.885204 | 5.896294 | 2 | 31218.67 | 19181.34 | 1.627555 | 3.842103 | 2 | 29085.43 | 20250.34 | 1.436294 | 4.229342 |
| 3 | 20568.36 | 41224.63 | 0.498934 | 3.32337 | 3 | 18311.24 | 23964.17 | 0.764109 | 1.803802 | 3 | 9445.903 | 22568.17 | 0.41855 | 1.232471 |
| 4 | 6054.539 | 32876.36 | 0.184161 | 1.226685 | 4 | 23622.43 | 40170.14 | 0.588059 | 1.388209 | 4 | 5095.752 | 32269.6 | 0.157912 | 0.464991 |

RAW data

**Figure 3C**

| | HMGB1 | Pon S | HMGB1/Pc | fold | | HMGB1 | Pon S | HMGB1/Pc | fold | | HMGB1 | Pon S | HMGB1/Pc | fold |
|---|---|---|---|---|---|---|---|---|---|---|---|---|---|---|
| 1 | 1626.284 | 24023.62 | 0.067695 | 1 | 1 | 2906.669 | 25190.45 | 0.115388 | 1 | 1 | 1323.778 | 21776.74 | 0.060789 | 1 |
| 2 | 15464.98 | 25343.57 | 0.610213 | 9.014128 | 2 | 21819.76 | 24210.45 | 0.901254 | 7.810655 | 2 | 20194.81 | 24375.86 | 0.828476 | 13.6288 |
| 3 | 8785.033 | 26803.15 | 0.327761 | 4.841719 | 3 | 8575.276 | 28667.47 | 0.299129 | 2.592382 | 3 | 3888.426 | 27542.93 | 0.141177 | 2.322423 |
| 4 | 12141.91 | 21608.33 | 0.561909 | 8.300572 | 4 | 13517.15 | 22622.74 | 0.597503 | 5.178218 | 4 | 10126.1 | 19580.55 | 0.517151 | 8.507369 |
| 5 | 19576.71 | 25266.81 | 0.774799 | 11.44541 | 5 | 16483.52 | 24618.86 | 0.669548 | 5.802595 | 5 | 22369.23 | 23536.4 | 0.95041 | 15.63467 |
| 6 | 1435.577 | 27618.45 | 0.051979 | 0.767837 | 6 | 3057.87 | 27682.52 | 0.110462 | 0.957312 | 6 | 1221.607 | 26093.4 | 0.046817 | 0.770156 |
| 7 | 1398.87 | 22948.45 | 0.060957 | 0.900463 | 7 | 3258.284 | 23026.28 | 0.141503 | 1.226325 | 7 | 1218.121 | 22765.03 | 0.053508 | 0.880237 |

RAW data

**Figure 3D**

| | HMGB1 | ponS | HMGB1/Pc | fold | | HMGB1 | ponS | HMGB1/Pc | fold | | HMGB1 | ponS | HMGB1/Pc | fold |
|---|---|---|---|---|---|---|---|---|---|---|---|---|---|---|
| 1 | 1551.184 | 11043.86 | 0.140457 | 1 | 1 | 1684.598 | 19085.62 | 0.088265 | 1 | 1 | 1213.163 | 10317.15 | 0.117587 | 1 |
| 2 | 24393.47 | 17459.28 | 1.397164 | 9.947294 | 2 | 23341.47 | 19362.15 | 1.20552 | 13.65792 | 2 | 21025.18 | 10069.5 | 2.088006 | 17.75712 |
| 3 | 3756.163 | 15005.5 | 0.250319 | 1.78218 | 3 | 9288.497 | 19760.91 | 0.470044 | 5.325353 | 3 | 3987.598 | 16712.45 | 0.2386 | 2.02914 |
| 4 | 9610.619 | 9026.376 | 1.064726 | 7.58046 | 4 | 16677.5 | 15408.38 | 1.082366 | 12.26264 | 4 | 11472.13 | 13591.91 | 0.844041 | 7.178016 |
| 5 | 20502.57 | 13072.62 | 1.56836 | 11.16615 | 5 | 19576.23 | 9239.761 | 2.118694 | 24.0037 | 5 | 20469.35 | 10008.08 | 2.045281 | 17.39377 |
| 6 | 2193.062 | 7703.619 | 0.284679 | 2.026813 | 6 | 1086.87 | 16906.3 | 0.064288 | 0.728348 | 6 | 3057.991 | 21984.1 | 0.1391 | 1.182955 |
| 7 | 578.163 | 12659.86 | 0.045669 | 0.325146 | 7 | 1031.506 | 12823.64 | 0.080438 | 0.911319 | 7 | 3262.841 | 20815.98 | 0.156747 | 1.333029 |

RAW data

**Figure 3E**

| | HMGB1 | ponS | HMGB1/Pc | fold | | HMGB1 | ponS | HMGB1/Pc | fold | | HMGB1 | ponS | HMGB1/Pc | fold |
|---|---|---|---|---|---|---|---|---|---|---|---|---|---|---|
| 1 | 1240.326 | 15611.64 | 0.079449 | 1 | 1 | 2165.426 | 17942.62 | 0.120686 | 1 | 1 | 1061.284 | 4487.033 | 0.236522 | 1 |
| 2 | 21209.76 | 18788.23 | 1.128886 | 14.20897 | 2 | 26080.88 | 16136.98 | 1.616218 | 13.39191 | 2 | 20031.98 | 7469.569 | 2.681812 | 11.33851 |
| 3 | 7678.912 | 16147.1 | 0.47556 | 5.985738 | 3 | 12297.91 | 16403.86 | 0.749696 | 6.211948 | 3 | 12792.21 | 7500.397 | 1.705537 | 7.210889 |
| 4 | 2510.64 | 9811.205 | 0.255895 | 3.220882 | 4 | 2382.991 | 13403.03 | 0.177795 | 1.4732 | 4 | 5817.426 | 4960.397 | 1.172774 | 4.958406 |
| 5 | 10051.62 | 16232.91 | 0.619212 | 7.793854 | 5 | 8233.012 | 18956.03 | 0.434321 | 3.598767 | 5 | 13035.86 | 7640.69 | 1.70611 | 7.213313 |
| 6 | 1077.355 | 14230.45 | 0.075708 | 0.952912 | 6 | 718.749 | 16505.21 | 0.043547 | 0.360827 | 6 | 2814.82 | 9858.569 | 0.28552 | 1.207159 |
| 7 | 762.113 | 14661.52 | 0.05198 | 0.654264 | 7 | 1192.163 | 6865.841 | 0.173637 | 1.438747 | 7 | 3087.577 | 9816.134 | 0.314541 | 1.329857 |
