# Supplementary material for: Formononetin inhibits lipopolysaccharide-induced release of high mobility group box 1 by upregulating SIRT1 in a PPARδ-dependent manner
Source: PeerJ. 2018 Jan 3;6:e4208. doi: 10.7717/peerj.4208 (PMC5756453; doi:10.7717/peerj.4208)
Supplement: Supplemental Information 4 — Raw and statistical data for the ratios of acetyl-HMGB1 to HMGB1 of Fig. 4A. Raw and statistical data for the ratios of acetyl-HMGB1 to HMGB1 of Fig. 4B. Raw and statistical data for the ratios of acetyl-HMGB1 to HMGB1 of Fig. 4C. [file peerj-06-4208-s004.pdf]

RAW data

Figure 4A

	LPS+Formononetin: acetyl-HMGB1					LPS+Formononetin: acetyl-HMGB1					LPS+Formononetin: acetyl-HMGB1			
	acetyl	HMGB1	acetyl/HM	fold		acetyl	HMGB1	acetyl/HM	fold		acetyl	HMGB1	acetyl/HM	fold
1	22568.54	32312.39	0.698449	1	1	8799.933	17545.9	0.501538	1	1	11558.27	17747.71	0.651254	1
2	55704.51	31089.54	1.791744	2.565321	2	35203.46	23311.97	1.510102	3.010944	2	40521.1	19485.9	2.079508	3.193083
3	18029.27	28616.2	0.630037	0.902052	3	27404.39	23960.32	1.143741	2.280468	3	12312.02	23001.61	0.535268	0.821904
4	18864.27	25998.13	0.725601	1.038876	4	17951.95	23624.97	0.759872	1.515084	4	7825.924	25252.61	0.309906	0.47586
5	8171.347	26476.25	0.308629	0.441878	5	6432.828	31211.34	0.206105	0.410947	5	6971.024	29254.87	0.238286	0.365888

RAW data

Figure 4B

	LPS+Formononetin: acetyl-HMGB1					LPS+Formononetin: acetyl-HMGB1					LPS+Formononetin: acetyl-HMGB1			
	acetyl	HMGB1	acetyl/HM	fold		acetyl	HMGB1	acetyl/HM	fold		acetyl	HMGB1	acetyl/HM	fold
1	1524.406	25267.98	0.06033	1	1	4848.033	21556.81	0.224896	1	1	5680.669	26512.52	0.214264	1
2	10067.18	26489.64	0.380042	6.299433	2	22010.95	22347.91	0.984922	4.379463	2	22096.64	23830.38	0.927247	4.327597
3	3297.861	22953.38	0.143677	2.381528	3	6119.79	23868.91	0.256392	1.140047	3	11758.1	24287.6	0.48412	2.259458
4	10239.1	29470.26	0.347439	5.759012	4	16027.64	23743.91	0.675021	3.001486	4	25993.35	26877.69	0.967098	4.513586
5	10906.71	23388.5	0.466328	7.729678	5	13797.57	23251.26	0.593412	2.63861	5	20848.23	23105.26	0.902315	4.211238
6	2010.134	22190.79	0.090584	1.501489	6	5885.083	21409.18	0.274886	1.222282	6	13886.98	28011.76	0.495755	2.313764
7	3869.518	28154.25	0.13744	2.278153	7	10857.47	29785.13	0.364527	1.62087	7	10031.31	23510.81	0.426668	1.991322

RAW data

Figure 4C

	acetyl-HM	HMGB1	acetyl/HM	fold		acetyl-HM	HMGB1	acetyl/HM	fold		acetyl-HM	HMGB1	acetyl/HM	fold
1	3900.719	27020.05	0.144364	1	1	1234.447	17489.35	0.070583	1	1	519.213	25612.4	0.020272	1
2	20891.93	25407.45	0.822276	5.695858	2	9015.832	17644.15	0.510981	7.239459	2	3991.267	26740.03	0.149262	7.362978
3	6306.154	24413.52	0.258306	1.789269	3	2437.912	26202.15	0.093042	1.318203	3	2098.74	26056.69	0.080545	3.973233
4	14012.23	23261.52	0.602378	4.172637	4	10682.25	24466.62	0.436605	6.185713	4	3541.64	24587.57	0.144042	7.105481
5	24391.13	24500.52	0.995535	6.896014	5	13673.83	22882.69	0.597562	8.466118	5	2813.861	24980.45	0.112643	5.556574
6	4221.548	23013.4	0.183439	1.270669	6	1183.841	20332.55	0.058224	0.824903	6	661.263	22215.96	0.029765	1.468297
7	1906.234	21155.52	0.090106	0.624157	7	928.255	24605	0.037726	0.534497	7	542.912	21162.81	0.025654	1.265496
