# Supplementary figures and images for: Formononetin inhibits lipopolysaccharide-induced release of high mobility group box 1 by upregulating SIRT1 in a PPARδ-dependent manner
Source: PeerJ. 2018 Jan 3;6:e4208. doi: 10.7717/peerj.4208 (PMC5756453; doi:10.7717/peerj.4208)

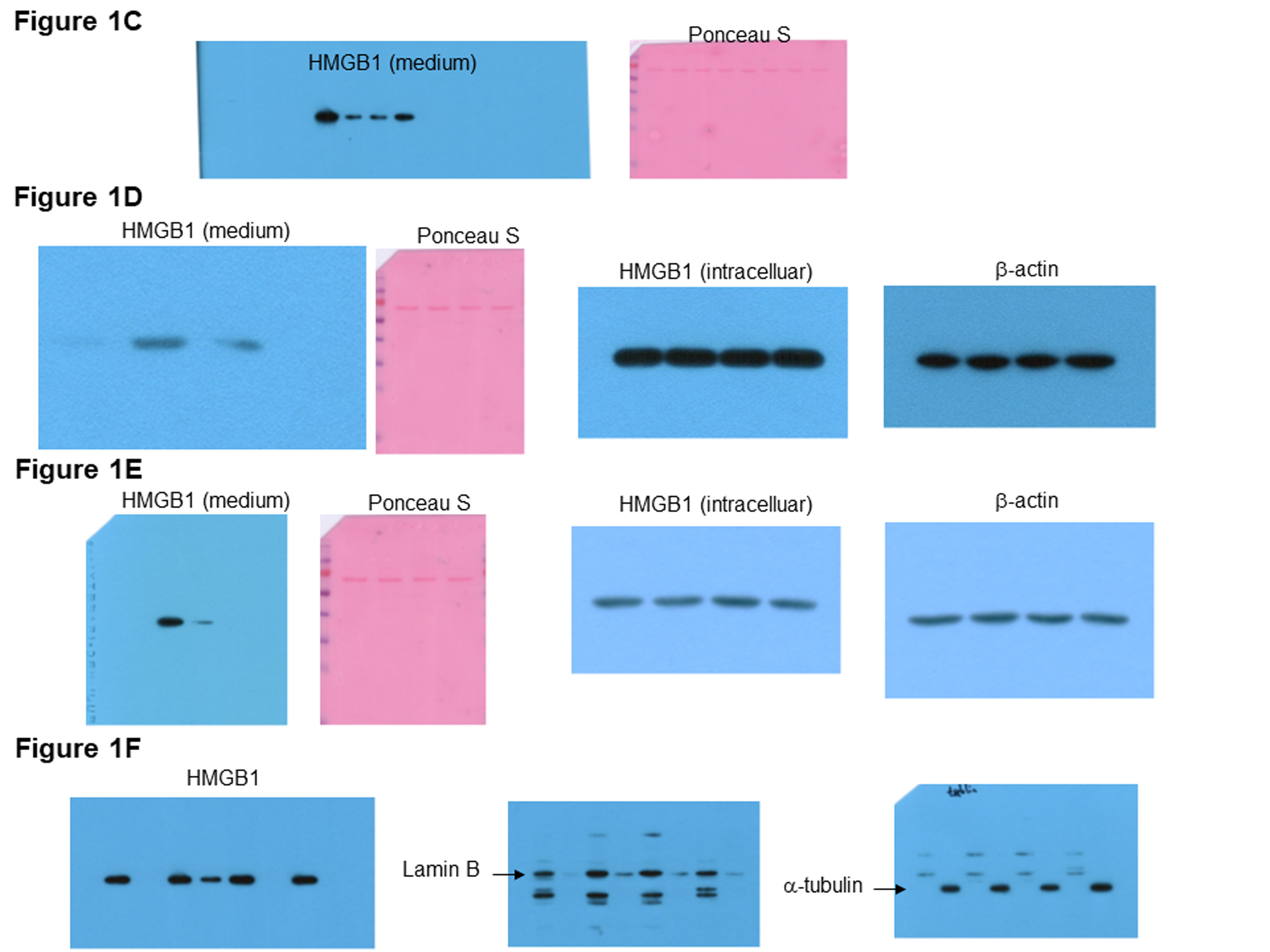

Supplement: Supplemental Information 5 — Uncropped blots for each figure. [file peerj-06-4208-s005.zip › WB raw data for Figure 1.PNG]

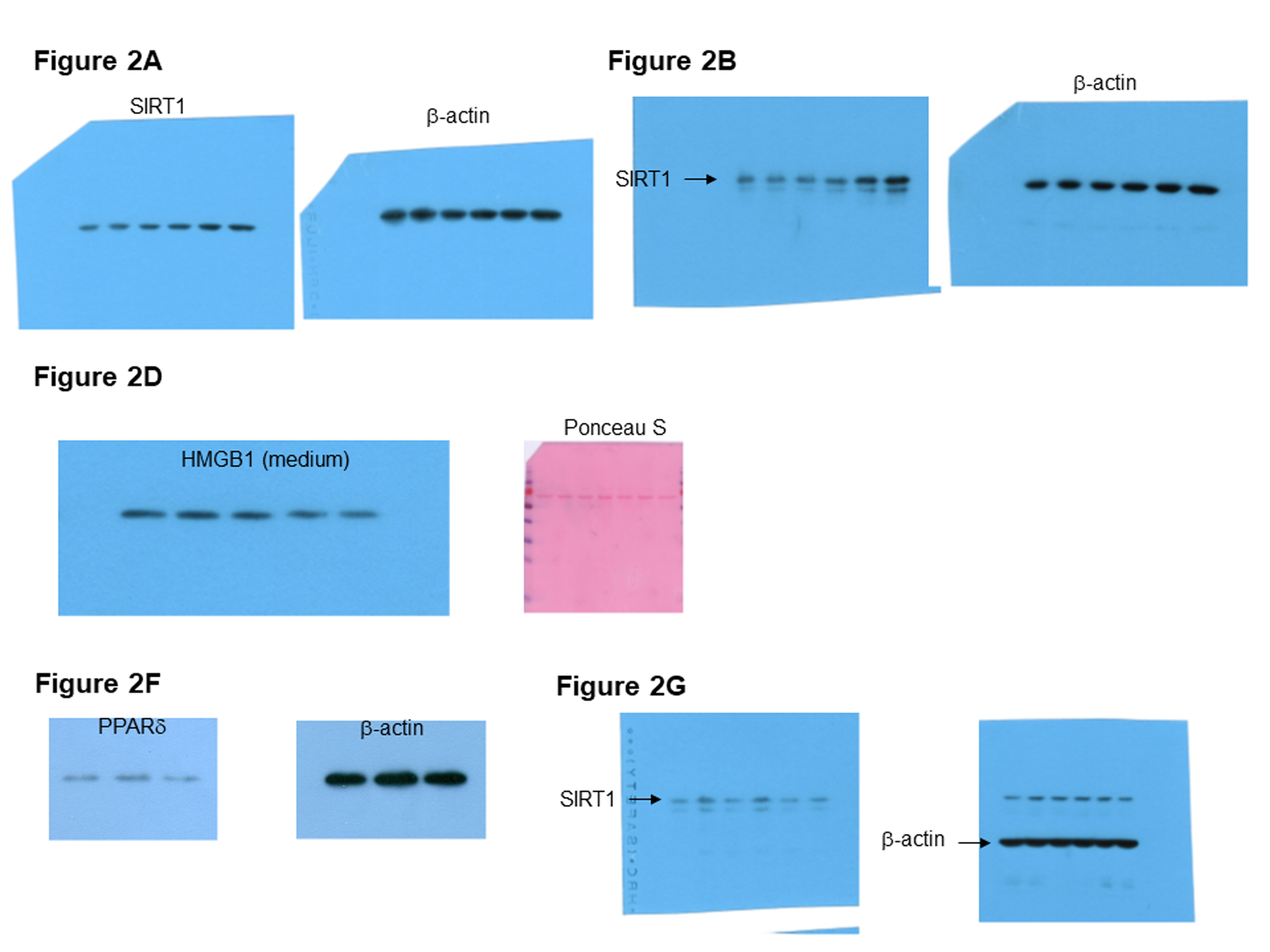

Supplement: Supplemental Information 5 — Uncropped blots for each figure. [file peerj-06-4208-s005.zip › WB raw data for Figure 2.PNG]

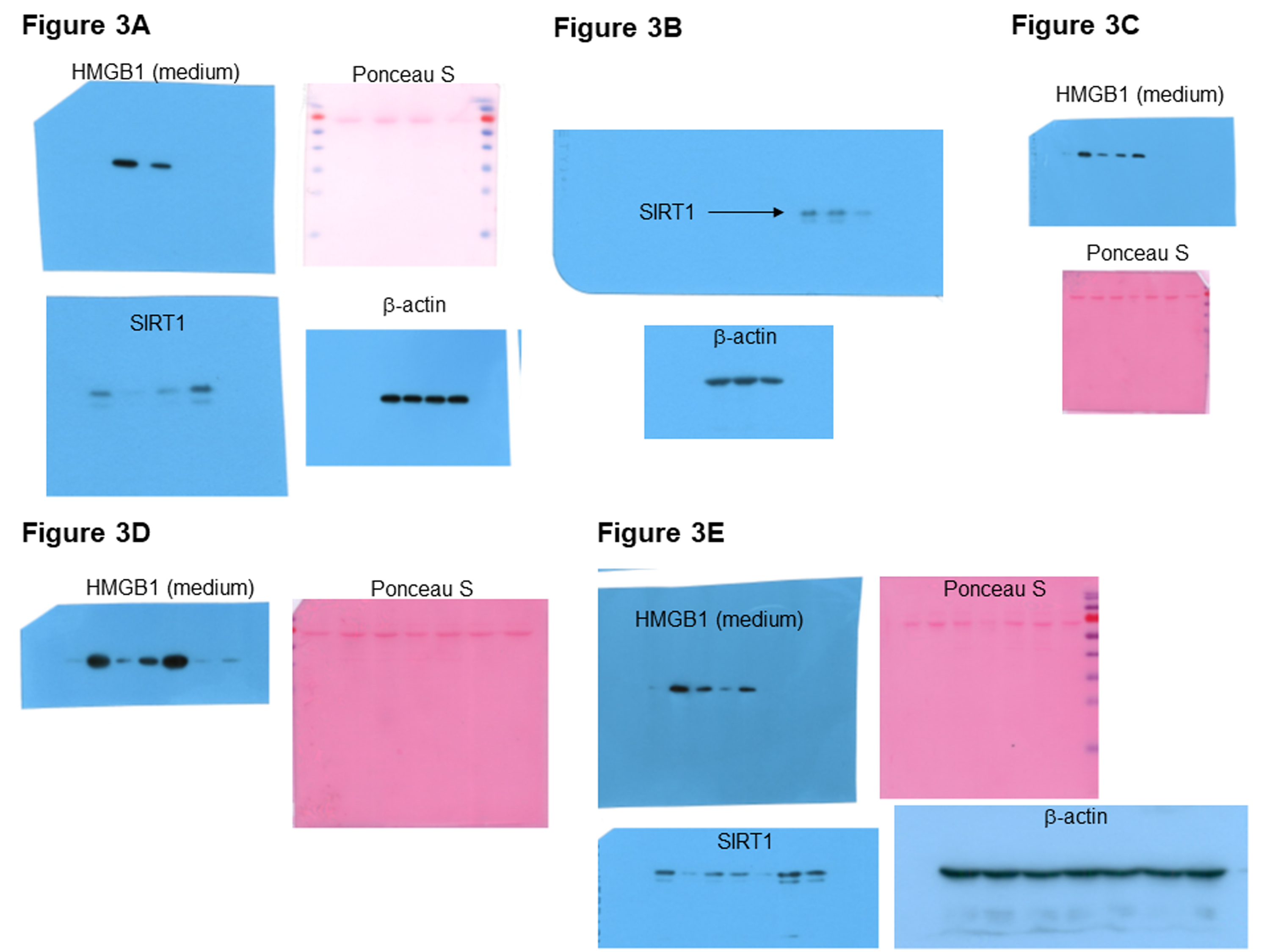

Supplement: Supplemental Information 5 — Uncropped blots for each figure. [file peerj-06-4208-s005.zip › WB raw data for Figure 3.PNG]

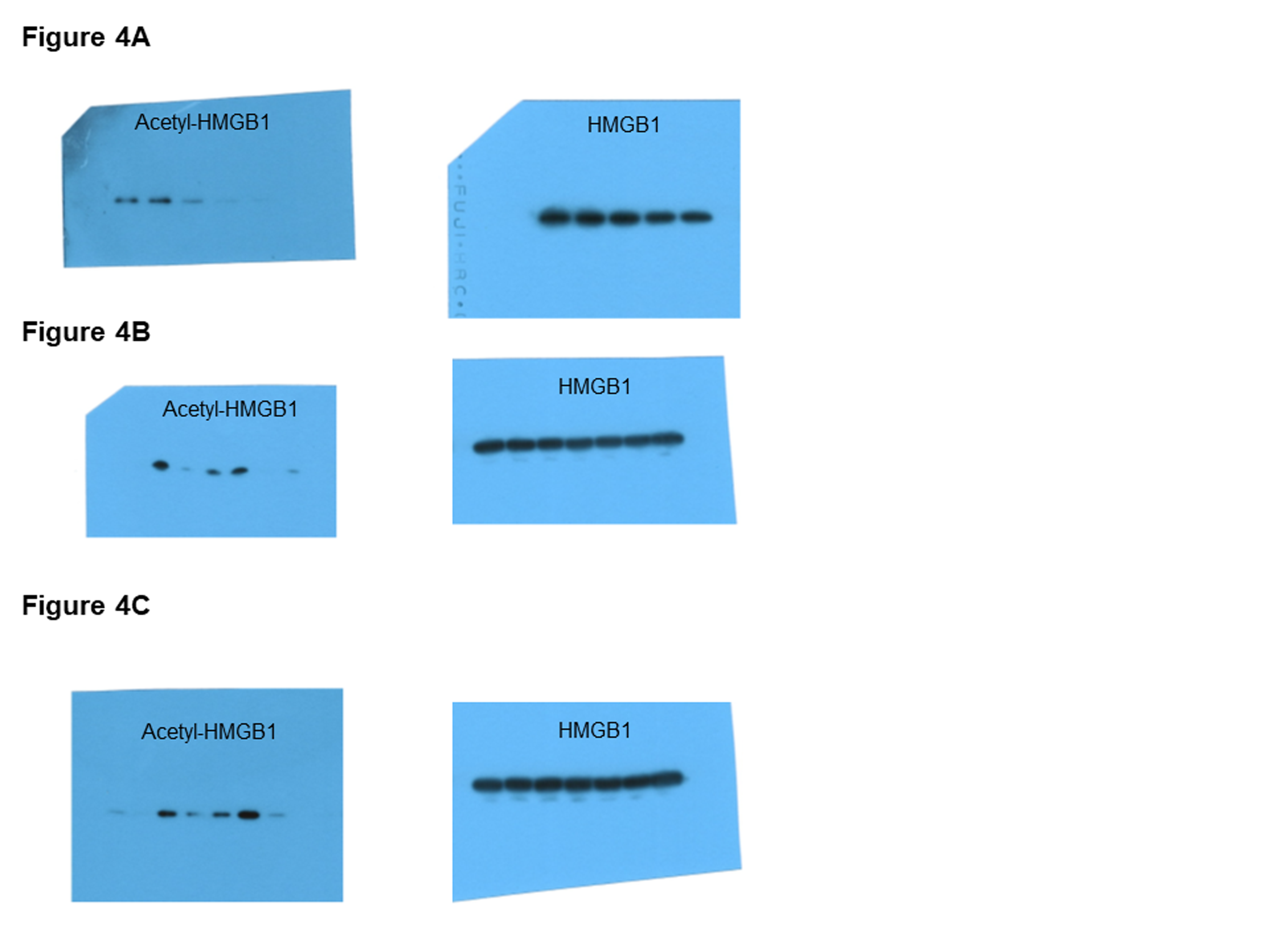

Supplement: Supplemental Information 5 — Uncropped blots for each figure. [file peerj-06-4208-s005.zip › WB raw data for Figure 4.PNG]
